# *Neisseria gonorrhoeae* Aggregation Reduces Its Ceftriaxone Susceptibility

**DOI:** 10.3390/antibiotics7020048

**Published:** 2018-06-15

**Authors:** Liang-Chun Wang, Madeline Litwin, Zahraossadat Sahiholnasab, Wenxia Song, Daniel C. Stein

**Affiliations:** Department of Cell Biology and Molecular Genetics, University of Maryland College Park, College Park, MD 20904, USA; mlitwin@terpmail.umd.edu (M.L.); nz.sahiholnasab@gmail.com (Z.S.); wenxsong@umd.edu (W.S.); dcstein@umd.edu (D.C.S.)

**Keywords:** gonorrhea, antibiotic resistance, increased susceptibility, biofilm, treatment failure, recurrence

## Abstract

Antibiotic resistance in *Neisseria gonorrhoeae* (GC) has become an emerging threat worldwide and heightens the need for monitoring treatment failures. *N. gonorrhoeae*, a gram-negative bacterium responsible for gonorrhea, infects humans exclusively and can form aggregates during infection. While minimal inhibitory concentration (MIC) tests are often used for determining antibiotic resistance development and treatment, the knowledge of the true MIC in individual patients and how it relates to this laboratory measure is not known. We examined the effect of aggregation on GC antibiotic susceptibility and the relationship between bacterial aggregate size and their antibiotic susceptibility. Aggregated GC have a higher survival rate when treated with ceftriaxone than non-aggregated GC, with bacteria in the core of the aggregates surviving the treatment. GC lacking opacity-associated protein or pili, or expressing a truncated lipooligosaccharide, three surface molecules that mediate GC-GC interactions, reduce both aggregation and ceftriaxone survival. This study demonstrates that the aggregation of *N. gonorrhoeae* can reduce the susceptibility to antibiotics, and suggests that antibiotic utilization can select for GC surface molecules that promote aggregation which in turn drive pathogen evolution. Inhibiting aggregation may be a potential way of increasing the efficacy of ceftriaxone treatment, consequently reducing treatment failure.

## 1. Introduction

Gonorrhea is the second most commonly reported sexually transmitted infection (STI) in the United States [1]. It is caused by *Neisseria gonorrhoeae* (GC), a gram-negative diplococcal bacterium. GC colonizes and infects the human genital tract but can also infect rectal and pharyngeal mucosal tissue in both men and women [2]. Symptoms of genital infection include painful urination, genital pain, and abnormal discharge. Nonetheless, the infection is often asymptomatic [3,4], with the asymptomatic rates as high as 56% in men [5] and 80% in women [6]. Asymptomatic infections cause extended colonization without treatment. Such untreated infections raise major concerns on the transmittance of gonorrhea and other STIs. In women, if left untreated, GC infection can lead to complications such as pelvic inflammatory disease (PID) and disseminated gonococcal infection (DGI) [7]. Consequences of PID include scarring of the reproductive organs, which may result in chronic pelvic pain, predisposition to ectopic pregnancy, and/or infertility. DGI can cause arthritis, tenosynovitis, dermatitis, and skin lesions [8]. The significance of gonorrhea is further highlighted by the findings that GC infection increases the risk of HIV infection and co-infections of other sexually transmitted pathogens [9].

Emerging antibiotic resistance of GC has become a public health crisis and a social-economic burden [10]. Declining susceptibility to cephalosporin resulted in a change in the treatment regimen from a single antibiotic to dual therapy, combining ceftriaxone with either azithromycin or doxycycline [11]. The emerging threat of cephalosporin resistance, in combination with subclinical gonorrhea, highlights the need for further understanding gonorrhea treatment failures.

The minimal inhibitory concentration (MIC) test has been the standard test for bacterial antibiotic resistance. However, whether this test reflects bacterial antibiotic resistance in vivo is unclear. For example, it has been well documented that pharyngeal gonococcal infections persist after antibiotic treatment [12]. Nonetheless, GC isolates from pharyngeal infections are sensitive to the antibiotics using the minimal inhibitory concentration test data. These findings suggest that unknown factors contribute to GC survival of antibiotic treatment in patients. Identifying these unknown factors might permit the development of more effective antimicrobial therapies.

The formation of bacterial biofilms has been shown to be a significant contributor to the survival of bacteria. Bacteria in biofilms are significantly more resistant to antimicrobials than bacteria in the planktonic phase of growth [13]. While GC can form biofilms [14], their contribution to antibiotic resistance and/or treatment failure in GC infections has not yet been examined. The GC biofilm progresses from an intimately associated aggregation of microcolonies to an organized biofilm. Pili, opacity-associated protein (Opa), and lipooligosaccharides (LOS), three well-characterized phase variable surface molecules of GC, have been shown to mediate inter-bacterium interactions to form aggregates [15,16,17]. How phase variations in their expression contribute to different GC-GC interactions and whether these interactions affect GC susceptibility to antibiotics is unknown. This study examines the role of GC aggregation, modulated by surface molecules, in bacterial survival in the presence of antibiotics. The reduced susceptibility of GC aggregates to antibiotics may contribute to treatment failure and recurrent gonorrhea.

## 2. Results

### 2.1. GC Aggregation Promotes Survival through Limiting Ceftriaxone Penetration

#### 2.1.1. GC Aggregation Promotes Survival under Ceftriaxone Treatment

**Figure 1:**
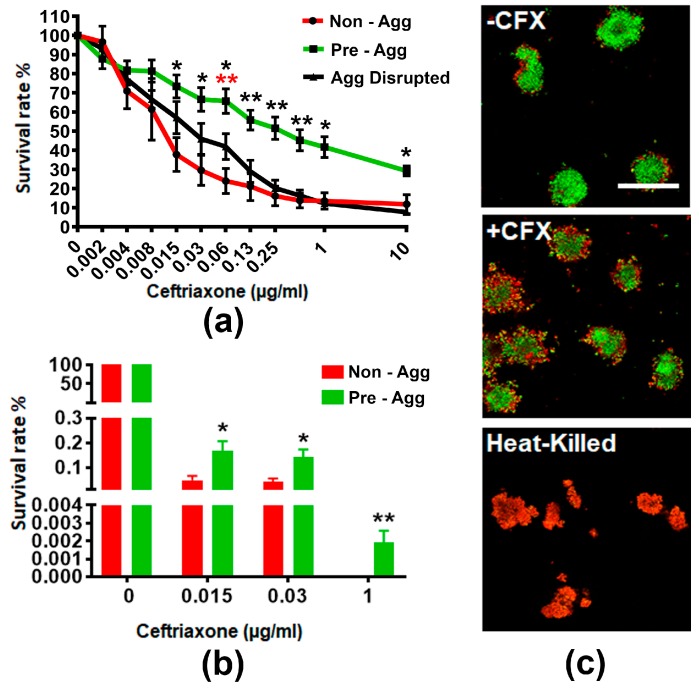
Survival rate and distribution of pre-aggregated MS11Opa+Pil+ *Neisseria gonorrhoeae* (GC) under ceftriaxone treatment. MS11Opa+Pil+ were inoculated in a 96-well plate and the suspensions were either not allowed to aggregate, allowed to pre-aggregate for 6 h, or allowed to pre-aggregate for 6 h before disrupting the suspension. Various concentrations of ceftriaxone were added into different aggregation conditions and incubated for 24 h. (**a**) BacTiter assay was performed to measure total ATP. (**b**) GC suspensions treated with 0, 0.015, 0.03, and 1 μg/mL ceftriaxone were plated onto a GCK plate. Colony forming units (CFU) were counted after 24 h incubation, and survival rates were calculated by dividing CFU from each condition to that with no ceftriaxone treatment. Shown are the average values (±SD) obtained from three independent experiments. *** *p* < 0.001; ** *p* < 0.01; * *p* < 0.05. (**c**) Pre-aggregated and heat-killed GC were incubated with or without 1 μg/mL ceftriaxone for 2 h, stained with Live/Dead BacLight stain to visualize viable (Green) and dead (Red) GC, and analyzed using a confocal fluorescence microscope. Shown are representative images from three independent experiments. +CFX: 1 μg/mL ceftriaxone; -CFX: mock control. Scale bar: 50 μm.

To examine if GC aggregation enhances resistance to ceftriaxone killing, we allowed a suspension of MS11Opa+Pil+ to either not aggregate, aggregate for 6 h, or aggregate for 6 h before disrupting the suspension. We then treated these aggregates with various concentrations of ceftriaxone for 24 h and measured the level of ATP production as an indication of GC viability by the BacTiter assay (Figure 1a). We found that the survival of non-aggregated, pre-aggregated, and aggregation-disrupted GC decreased as the concentration of ceftriaxone increased. However, the percentage of survival of pre-aggregated GC was significantly higher than non-aggregated and aggregation-disrupted GC when ceftriaxone concentrations approached the broth MIC of 0.125 µg/mL. To verify the results from the BacTiter assay, we quantified the cultivable CFU with and without pre-aggregation for 6 h in the absence or presence of 0.015, 0.03, and 1 µg/mL ceftriaxone (Figure 1b). Pre-aggregated and non-aggregated GC showed significant differences in the ATP production at these concentrations of ceftriaxone. We found the total cultivable CFU decreased >100 fold at both 0.015 and 0.03 µg/mL ceftriaxone and >10,000 fold at 1 µg/mL ceftriaxone compared to non-treatment control. Compared to non-aggregated GC, the number of cultivable CFU of pre-aggregated GC increased significantly at 0.015 and 0.03 µg/mL ceftriaxone. Furthermore, at 1 µg/mL ceftriaxone, there were still cultivable GC from the pre-aggregated group whereas non-aggregated GC had no growth. These data suggest GC aggregation promotes survival in the presence of ceftriaxone.

#### 2.1.2. GC Aggregation Limits Ceftriaxone Penetration

To investigate how aggregation enhances GC survival in the presence of antibiotics, we examined the distribution of live and dead GC within aggregates. We allowed MS11Opa+Pil+ to pre-aggregate for 6 h, treated with or without 1 µg/mL ceftriaxone for 2 h, and stained the resulting cultures with BacLight viability stain with heat-killed aggregates as our control (Figure 1c). Our confocal fluorescent microscopy analysis showed that after ceftriaxone treatment, dead bacteria (red) were mostly located at the outer layers whereas viable GC (green) were mostly located in the core of the MS11Opa+Pil+ aggregates. Taken together, our results indicate that GC aggregation reduces susceptibility to antibiotic treatment by limiting antibiotic penetration.

### 2.2. GC Strains Lacking Opa or Pili or Expressing Truncated LOS Showed a Reduced Survivability against Ceftriaxone Due to Decreased Aggregation

**Figure 2:**
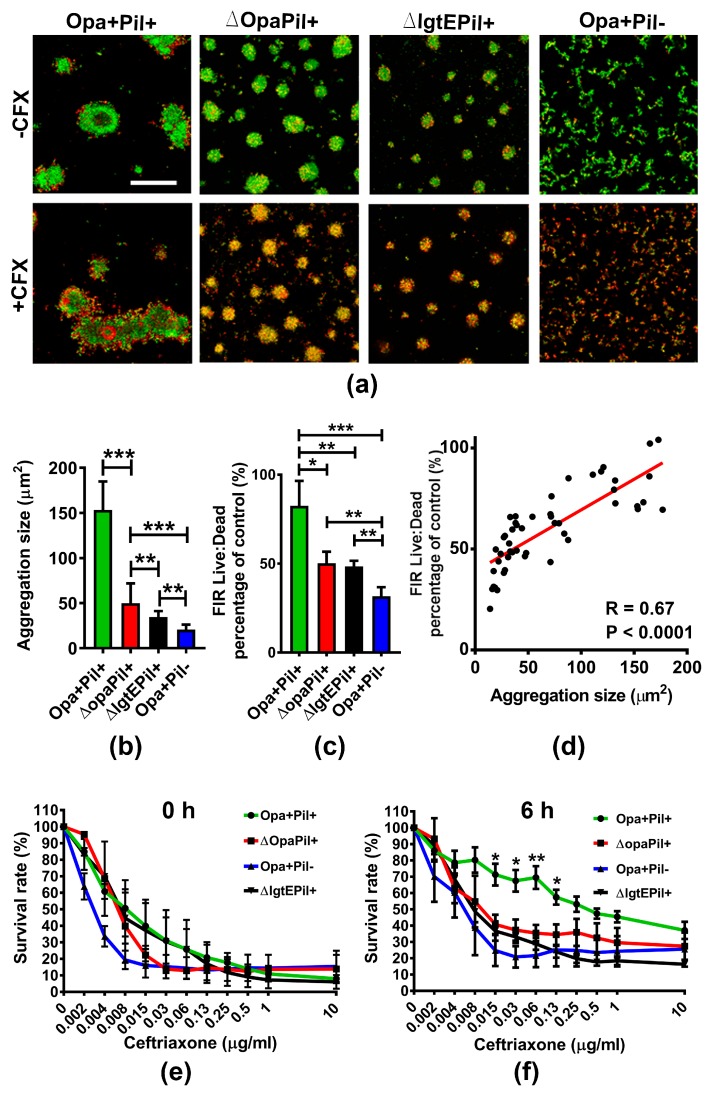
Comparison of survival rate and distribution in GC aggregates that lack Opa or Pili or express truncated lipooligosaccharides (LOS) under ceftriaxone treatment. MS11Opa+Pil+, MS11ΔOpaPil+, MS11Opa+Pil-, or MS11ΔLgtEPil+ were allowed to aggregate for 6 h. (**a**) Pre-aggregated GC were incubated with 1 μg/mL ceftriaxone (+CFX) or mock treated (-CFX) for 2 h, stained with Live/Dead BacLight stain for visualizing live (Green) and dead (Red) GC, and analyzed using a confocal fluorescence microscope. Scale bar: 50 μm. (**b**) The sizes of GC aggregates were quantified by the area of each aggregate. (**c**) The live to dead GC ratios were quantified by the fluorescence intensity ratio (FIR) of green to red staining. The average size of aggregation and live to dead ratio (±SD) were obtained from >40 images of three independent experiments. (**d**) The relation between GC aggregation and their susceptibility to antibiotics was analyzed by plotting the sizes of GC aggregates versus their survival rates and linear regression. (**e**,**f**) 0 h non- and 6 h pre-aggregated GC were incubated with serial concentrations of ceftriaxone for 24 h. The ATP production under each condition was measured. Shown are the average values (±SD) obtained from three independent experiments. *** *p* < 0.001; ** *p* < 0.01; * *p* < 0.05.

Opa, pili, and LOS, three major surface molecules of GC, play critical roles in promoting GC-GC interactions. To further investigate the role of GC aggregation in antibiotic susceptibility, we compared GC that switch off pili expression (Pil-), GC that had all 11 *opa* genes deleted (ΔOpa), or GC that cannot express the terminal Lacto-*N*-tetrose (ΔLgtE) with the wildtype phase variable strain. We allowed MS11Opa+Pil+, MS11ΔopaPil+, MS11Opa+Pil- and MS11ΔLgtEPil+ to pre-aggregate for 6 h and treated aggregates with or without 1 µg/mL ceftriaxone for 2 h. We then stained the resulting cultures with BacLight and analyzed the bacteria using confocal fluorescence microscopy (Figure 2a). We quantified the size of GC aggregates by measuring the area of individual aggregate and the survival rate of GC by determining the fluorescence intensity ratio (FIR) of live to dead bacteria in GC aggregates. Our images show that MS11Opa+Pil+ formed the largest and MS11Opa+Pil- formed smallest aggregates among all strains tested. The lack of Opa, complete LOS, or pili progressively and significantly reduced the size of GC aggregates (Figure 2a,b). GC in the outer layers of the large MS11Opa+Pil+ aggregates were dead while those in the core were still alive. In contrast, most of GC in the small loose aggregates of MS11ΔOpaPil+, MS11Opa+Pil-, and MS11ΔLgtEPil+ were mostly dead. Interestingly, MS11Opa+Pil- and MS11ΔLgtEPil+, forming similar sizes of aggregation, displayed a mixed distribution of live and dead GC; note the presence of yellow GC, indicating the uptake of both dyes (Figure 2a). We then compared the live to dead FIR of treated GC to those of untreated GC to measure the percentage of survival. MS11Opa+Pil+ had the highest surviving rate while MS11Opa+Pil- had the lowest among the four strains. Compared to MS11Opa+Pil+, the surviving levels of MS11ΔopaPil+ MS11ΔLgtEPil+, and MS11Opa+Pil- all were significantly decreased (Figure 2c). By plotting the number of GC surviving with the size of GC aggregates, we found a positive correlation (R = 0.67) between the two, with statistical significance (Figure 2d). We further confirmed this finding by measuring the ATP production. We found that without aggregation, all of the strains had similar and decreased levels of ATP (Figure 2e). However, with aggregation, MS11Opa+Pil+ aggregates had the highest ATP level post ceftriaxone treatment (0.015–0.125 µg/mL) while other strains significantly decreased the levels of ATP (Figure 2f). Taken together, these results indicate that the ability of GC surface molecules to promote aggregation is responsible for the decreased susceptibility of GC to ceftriaxone treatment.

### 2.3. GC Aggregation on Human Epithelial Cells also Increases Ceftriaxone Survivability

**Figure 3:**
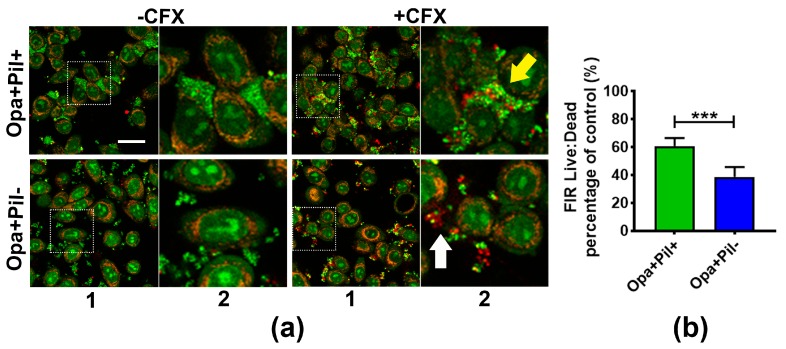
Survival and distribution of GC aggregates on human cervical epithelial cells. MS11Opa+Pil+ or MS11Opa+Pil- were inoculated and incubated with ME180 cells for 6 h. (**a**) GC- epithelial cell co-cultures were treated with 1 μg/mL ceftriaxone (+CFX) or mock treated (-CFX) for 2 h, stained with Live/Dead BacLight stain for visualizing live (Green) and dead (Red) GC, and analyzed using a confocal fluorescence microscope. The images shown in panel (1) reflect the overall distribution of cells and the image in panel (2) is an enlargement of the inset, shown as a dotted box. Yellow arrow, a large aggregate of Opa+Pil+ GC; White arrow, a small aggregate of Opa+Pil- GC. (**b**) The live to dead GC ratios were quantified by the FIR of green to red staining. The average ratio (±SD) was obtained from >30 images of three independent experiments. *** *p* < 0.001; ** *p* < 0.01; * *p* < 0.05. Scale bar, 30 μm.

To determine if GC aggregation also increases the antibiotic survivability of GC that are associated with human epithelial cells, we compared the survival of Opa+Pil+ and Opa+Pil- GC that were pre-incubated with the human cervical epithelial cells ME180. We incubated ME180 cells with MS11Opa+Pil+ or MS11Opa+Pil- for 6 h to let GC attach to epithelial cells and treated the co-culture with or without 1 µg/mL ceftriaxone for 2 h. The resulting cultures were stained with BacLight dye and analyzed by confocal fluorescence microscopy (Figure 3a). Similar to pre-aggregated GC in the absence of epithelial cells, MS11Opa+Pil+ formed larger and tighter aggregates than MS11Opa+Pil-. In ceftriaxone treated MS11Opa+Pil+ aggregates, only bacteria in the outer layer were dead whereas those in the core of the aggregates and at the side of the aggregates that attached to ME180 cells remained viable (yellow arrow). In the small aggregates of MS11Opa+Pil-, live and dead GC were mixed together (white arrow) no matter if they attached to ME180 cells or not. By comparing the live to dead FIRs, we found that after attaching to ME180 cells, MS11Opa+Pil+ still had a higher surviving rate of antibiotic treatment than MS11Opa+Pil- (Figure 3b), similar to what we observed in the absence of epithelial cells. These results support that aggregation helps GC that colonize human epithelial cells to survive ceftriaxone treatments. 

## 3. Discussion

The results of this study demonstrate that the ability to form aggregates significantly increased GC survival in the presence of ceftriaxone. Furthermore, the expression of GC surface molecules, Opa, pili, and LOS, enhances GC survival by promoting bacterial aggregation. While there is accumulating evidence for the importance of gonococcal aggregation in infection, this study is the first to reveal the contribution of GC aggregation to its reduced susceptibility to ceftriaxone.

The association of GC aggregation with their survivability in the presence of ceftriaxone suggests an additional mechanism by which GC increase their resistance to clinically relevant antibiotics. GC aggregation has been observed in patient biopsies and exudates where GC aggregates were found on ectocervical epithelium from displaying cervicitis and phagocytic immune cells from patients displaying urethritis [18,19]. Our ex vivo infection study using endocervical tissue explants found GC aggregates on the endocervical epithelium and GC biofilm on the ectocervical epithelium [20,21]. We also noticed that GC form aggregates not only on the surface of epithelium but also on exfoliated cervical epithelial cells even in the absence of Opa or pili expression (data not shown), suggesting the presence of unknown host factors that facilitate GC aggregation. Thus, GC aggregation and the increased antibiotic susceptibility of GC aggregation are likely to happen in vivo.

The surface molecules Opa, pili, and LOS are well-known for their roles in GC pathogenesis [22], but their roles in GC survival with antibiotic treatment have not been examined. This study shows that these molecules decrease GC susceptibility to antibiotic treatment mainly by forming aggregates through mediating bacterium-bacterium interaction. However, Opa, pili, and LOS also interact with host epithelial cells during infections in addition to mediating bacterium-bacterium interactions. GC aggregation and adherence to host cells through these molecules may influence each other, therefore changing both the infectivity and antibiotic resistance of GC in the reproductive tract of women. Indeed, we have previously shown that Opa expression facilitates GC adherence to host cells and GC aggregation, but reduces GC penetration into both polarized epithelial cell monolayers and the endocervical epithelium in the tissue explant model [16,20]. The functions of Opa in both GC-GC and GC-epithelial interactions enable Opa-expressing GC to survive better in the presence of antibiotic agents and dominate the colonization of the epithelial surface. 

Analysis of *opa* and *pil* genes across geographical locations, species, and strains suggests that the host immune system drives sequence polymorphisms within these variable genes [23]. Based on the results presented here, we can extend this model by postulating that antibiotic therapy applies an additional pressure on the gonococcus to select variants expressing surface molecules that promote inter-bacterial adhesion. Combining previously published findings and our results presented here together allows us to propose a working model: the more invasive but less aggregated GC are more susceptible to antibiotic killing. Antibiotic utilization will select for GC that can form strong GC-GC interactions but are less invasive. This model can also be correlated with the fact that DGI strains have been found to be hyper-susceptible to penicillin [24,25]. Our data also predict that as treatment failures accumulate, the resulting strains will be less able to cause disseminated/invasive diseases. From an epidemiological perspective, we should see an increase in the incidence of asymptomatic infection and a decrease in pelvic inflammatory disease, which are currently happening (4, 5 https://www.cdc.gov/std/stats16/womenandinf.htm#pid). 

In addition to aggregation, GC may also escape from antibiotic killing by invading into host cells. In men, it is suggested that GC use asialoglycoprotein receptor to invade into urethral epithelial cells [26]. In females, GC preferentially use human complement protein CR3 to adhere and invade ecto- and endo-cervix [27]. GC have also been shown to be capable of surviving and replicating inside neutrophils in vivo and in vitro [28], and the expression of different isoforms of Opa impacts GC survival fitness within neutrophils [29]. Moreover, penetrated GC may also invade into fibroblasts in order to escape contact with antibiotics*.* Grassme et al. have shown that GC can invade into human fibroblasts through Opa binding to heparan sulfate proteoglycans [30].

Taken together, the multifunctional roles of Opa, pili, LOS molecules in GC aggregation and invasion into host cells maximize the chance of GC survival of antibiotic treatment. Inhibiting the interactions of Opa, pili, and LOS with each other as well as with host cells potentially increase antibiotic treatment efficacy, thereby reducing the frequency of treatment failure and recurrence.

## 4. Materials and Methods

### 4.1. Bacteria Strains

*N*. *gonorrhoeae* strain MS11 that expressed phase-variable Opa and pili (MS11Opa+ Pil+) was obtained from Dr. Herman Schneider, Walter Reed Army Institute for Research. MS11ΔOpa and MS11ΔLgtE were previously described [17,31]. Pili negative colonies were identified based on colony morphology using a dissecting light microscope. GC was grown on plates with GC media (Difco, BD Bioscience, Franklin Lakes, NJ, USA) and 1% Kellogg’s supplement [32] at 37 °C with 5% CO_2_ for 16–18 h before use in experiments.

Epithelial cell line—ME180 cells, a human cervical epidermal carcinoma cell line (ATCC# HTB-33), were maintained in RPMI1640 supplemented with 10% heat-inactivated fetal bovine serum and 1% Penicillin-Streptomycin. ME180 cells were seeded at 1 × 10^5^ into 12 mm diameter round glass coverslips (VWR, Radnor, PA, USA) in a 24 well plate (Corning, Lowell, MA, USA) and incubated at 37 °C with 5% CO_2_. After 24 h, cells were switched into antibiotic-free medium overnight for aggregation assay.

### 4.2. BacTiter Assay

GC suspended in GC media with Kellogg’s supplement and NaHCO_3_ were seeded in a 96 well plate (10^7^ in 99 µL per well). The GC suspension was allowed to either not aggregate, aggregate for 6 h, or aggregate for 6 h before being disrupted by vortex. Serial dilutions of ceftriaxone (1 µL aliquots) were added into each well within each aggregation condition and incubated for 24 h. An equal volume of BacTiter glow reagent (Promega, Madison, WI, USA) was then added and incubated for 15 min. Optical absorbance was determined at the wavelength 560 nm using a Glomax Illuminamintor (Promega, Madison, WI, USA), and the survival rate was calculated by the ratio of the reading obtained after antibiotic treatment to the reading from untreated wells.

### 4.3. Fluorescence Microscopic Analysis of Live and Dead Bacteria in Aggregates

GC (10^7^/mL/well) were incubated in 8-well coverslip-bottom chambers (Sigma, St. Louis, MO, USA) or on ME180 cells in the coverslip for 6 h to allow the bacteria to form aggregates. Aggregated bacteria were treated with or without 1 µg/mL ceftriaxone for 2 h, or heat-killed at 65 °C for 15 min. These aggregates were then stained with Live/Dead BacLight Stain (Life Technology, Frederick, MD, USA) for 15 min. Z-series images were acquired using a confocal microscope (Leica SP5X). Images were analyzed using NIH ImageJ software to measure the size of GC aggregates and the fluorescence intensity ratio (FIR) of live to dead staining in each aggregate.

### 4.4. Statistical Analysis

Data were plotted and statistically analyzed using the two-tailed Student’s *t*-test and Linear Regression by Prism software (GraphPad Software, La Jolla, CA, USA).

## 5. Conclusions

This study demonstrates that the aggregation of *N. gonorrhoeae* can reduce the susceptibility to antibiotics, and suggests that antibiotic utilization can select for surface molecules that promote aggregation. This can drive pathogen evolution for better colonization but with reduced invasive capabilities. Moreover, inhibiting aggregation may be a potential way of increasing the efficacy of antibiotic treatment, consequently reducing treatment failure.
